# Differential infiltration of CD4+ and CD8+ T cells and expression of PD-L1 in paired biopsy and resection specimens of gastric and colorectal adenocarcinomas

**DOI:** 10.3389/fonc.2026.1830997

**Published:** 2026-06-22

**Authors:** Zi-xuan Wang, Chun-mei Zhang, Yao-zhen Liu, Yu-wei Zhang, Shi-jie Tong, Qi Liu, Duo Deng, Yun Pan

**Affiliations:** 1 School of Basic Medical Sciences, Dali University, Yunnan, China; 2 Department of Pathology, First Affiliated Hospital of Dali University, Yunnan, China

**Keywords:** biopsy and resection, CD4+ T cells, CD8+ T cells, immune microenvironment, PD-L1

## Abstract

**Objective:**

To compare CD4+ T cell, CD8+ T cell infiltration and PD-L1 expression between paired biopsy and resection specimens in gastric and colorectal adenocarcinoma, and evaluate their clinical significance.

**Methods:**

Paired biopsy and resection specimens from 38 gastric and 40 colorectal adenocarcinoma patients were assessed by immunohistochemistry for CD4+ T cell, CD8+ T cell density and PD-L1 expression. Correlations with clinicopathological parameters and tumor markers were analyzed.

**Results:**

In gastric adenocarcinoma, resection specimens showed significantly higher CD4+ T cell, CD8+ T cell density and PD-L1 expression than biopsies (all P<0.05), with CD4+ T cells positively correlated with CA19-9 (R = 0.523, P = 0.026). In colorectal adenocarcinoma, CD8+ T cell density was higher in resection specimens (P = 0.0353); CD4+ T cells negatively correlated with Ki-67 (R=-0.370, P = 0.019), and CD4+/CD8+ ratio negatively correlated with mismatch repair protein expression (R=-0.342, P = 0.029). In both cancers, CD4+/CD8+ ratios at tumor center and invasive margin were positively correlated (gastric: R = 0.5683, P = 0.0002; colorectal: R = 0.7324, P<0.0001). Preoperative neutrophil-to-lymphocyte ratio positively correlated with tumor diameter in both cancers (gastric: R = 0.449, P = 0.011; colorectal: R = 0.631, P = 0.001). CD4+ T cell density differed significantly between cancer types (P <0.0001). ROC analysis demonstrated limited predictive value of biopsy specimens for surgical findings, particularly in colorectal cancer (AUC 0.5-0.7).

**Conclusion:**

Biopsy specimens inadequately represent the immune microenvironment of resection specimens, especially in gastric adenocarcinoma. Tumor-type-specific immune assessment of biopsies is essential for guiding precise immunotherapy decisions.

## 1 Introduction

Gastrointestinal (GI) cancers, including gastric cancer (GC) and colorectal cancer (CRC), account for 26% of global cancer cases and 35% of cancer-related deaths (1). Gastric cancer represents 5.6% of cases and 7.7% of deaths, ranking among the world’s top five in both incidence and mortality. Gastric cancer is a heterogeneous disease, with adenocarcinoma being the primary histological subtype, accounting for 90% of all gastric cancers (2). CRC accounts for 10% of cases and 9.4% of deaths, making it the third most common cancer and fourth leading cause of cancer mortality (3, 4). More than 90% of colorectal cancers are adenocarcinomas, originating from the epithelial cells of the colorectal mucosa (5). Surgery is the only curative option for gastric cancer, with a >90% 5-year survival rate in early stage cases. However, over 70% of patients are diagnosed at advanced stages, reducing survival to <20% (6). Similarly, treatment options such as laparoscopic surgery for colorectal cancer primary foci, surgical resection for metastatic foci, and chemoradiotherapy have limited impact on the cure rate and long-term survival of colorectal cancer patients, with the 5-year survival rate of patients being less than 10 percent (7).Immunotherapy offers new hope for GI cancer patients (8). The tumor immune microenvironment (TIME) plays a critical role in cancer progression, and a deeper understanding of immune cells interactions is essential to identify novel therapeutic targets and improve treatment efficacy (9, 10).

The tumor microenvironment (TME) is a complex ecosystem composed of immune cells, stromal cells and extracellular matrix (11). Among them, tumor-infiltrating lymphocytes (TILs), as the core executors of anti-tumor immune response, their number, subpopulation distribution, and functional status directly affect the immune escape and therapeutic response of tumor (12). Furthermore, studies have shown that TILs can serve as prognostic and predictive markers of immunotherapy response in cancer patients (13). Clinical evidence suggests that the degree of TILs infiltration is significantly associated with patient prognosis Zhang et al. indicate that after radical surgery, patients with high TILs tumors have better disease-free survival (DFS) and overall survival (OS) than patients with low TILs, suggesting that TILs-mediated immune responses inhibit tumor recurrence (14). Mechanistically, antitumorigenic CD8+ TILs directly induce apoptosis in tumor cells by releasing granzyme B and perforin, while secreting IFN-γ and TNF-α to inhibit proliferation and enhance antigen presentation (15). However, tumors can escape attack by TILs through the immune checkpoint pathway. Programmed death receptor 1 (PD-1) is a co-inhibitory molecule that is physiologically expressed on the surface of T cells, B cells, and myeloid cells and is used to terminate the immune response (16, 17). PD-1 is highly expressed on the surface of TILs under sustained antigenic stimulation, and tumor cells inhibit T cells proliferation and cytotoxicity by binding to it via PD-L1 and reducing granzyme B and cytokine IFN-γ secretion (18). Therefore, targeting the PD-L1/PD-1 axis can lift this inhibition and restore the immunosurveillance function of TILs (19).

Immune check‐point inhibitors (ICIs) have emerged as a cornerstone of oncological therapy by reactivating antitumor immune responses through the blockade of immune checkpoint molecules such as PD-1, PD-L1, and CTLA-4, and ICIs have received regulatory approval in numerous countries for a wide range of tumor types and clinical indications (20). TME characteristics, including TILs density and PD-L1 expression status, serve as prognostic markers for predicting recurrence, disease-free survival and overall survival in gastric and colorectal cancers (21). In addition, TME characteristics are increasingly recognized as predictive biomarkers of immunotherapy response in various malignancies (22). Research confirms that in gastric cancer, high-density CD8+ T cells infiltration, particularly when coexisting with high PD-L1 expression, serves as a marker for favorable prognosis and predictive of good response to immunotherapy (23). Meanwhile, in colorectal cancer, MicroSatellite Instability-High (MSI-H) serves as a potent predictive biomarker (24). MSI-H patients derive substantial benefit from immunotherapy. Thierry André et al. confirmed that MSI-H patients exhibit high sensitivity to immunotherapy, whereas MicroSatellite Stable (MSS) patients show minimal efficacy (25). Dung T Le et al. demonstrated that mismatch repair deficiency (dMMR), which leads to MSI-H colorectal cancer, exhibits a higher response rate to PD-1 inhibitors. This is because these tumors harbor a large number of TILs (26). With the increasing popularity of neoadjuvant immunotherapy trials, it is important to incorporate TME assessment into clinical guidelines and to ensure accurate pre-treatment assessment using biopsy and resection specimens (27). However, about 70% of gastric cancer patients and 30% of colorectal cancer patients are in locally progressive or metastatic stage (Stage III/IV) at the time of initial diagnosis, and are unable to obtain resection specimens through surgery, and have to rely on biopsy (3). The study confirmed that the correlation of TIL subpopulations (e.g., CD8+ T cells) was weaker in biopsy specimens compared with resection specimens, and there was significant heterogeneity in the spatial distribution of PD-L1 expression and TILs (28), with an average of 0.1-0.3 cm^3^ of biopsy specimens failing to reflect the full extent of the TME, suggesting that reliance on biopsy may fail to correctly assess the immune microenvironment for TILs and PD-L1, leading to incorrect therapeutic decisions (29).

The aim of this study was to investigate the differences and clinical significance of CD4+ and CD8+ T cell infiltration densities and PD-L1 expression between biopsy and resection specimens of gastric and colorectal cancer, to analyze the correlation of TILs infiltration in cancer and paracancerous tissues, and to explore the differences in TILs infiltration and PD-L1 expression between gastric and colorectal adenocarcinoma. Furthermore, we investigated the relationship between CD4, CD8, or PD-L1 expression and various tumor markers as well as clinicopathological features (including HER2, Ki67, etc.), aiming to elucidate the influence of the immune microenvironment on tumor progression and malignancy.

## 2 Methods

### 2.1 Patients and specimens

We conducted a retrospective study of 38 gastric and 40 colorectal adenocarcinoma patients from the First Affiliated Hospital of Dali University. All cases were histopathologically confirmed as adenocarcinoma. For each adenocarcinoma case, we collected matched tissue samples: biopsy specimens (average of 0.1-0.4 mm^3^), resection specimens, and matched paracancerous tissue obtained from areas >5 mm beyond the tumor margin. Patients who received neoadjuvant therapy prior to resection were excluded from the analysis. The interval between biopsy and surgical resection was recorded for all patients. In gastric cancer patients, the biopsy-to-resection interval ranged from 1 to 22 days (median 8 days); in colorectal cancer patients, the interval ranged from 4 to 12 days (median 7 days). Data collected for analysis included patient ethnicity, survival, tumor size, TNM stage, number of chemotherapy treatments and drugs, tumor markers, and preoperative and postoperative NLR of the patients.

In this study, HER2 status was evaluated by immunohistochemistry (IHC) according to the NCCN Clinical Practice Guidelines for Colon Cancer (Version 2.2025). IHC 0 was defined as no membranous staining; IHC 1+ (negative) as faint, incomplete membranous staining (segmental or granular) in any proportion of tumor cells, or moderate complete staining in <50% of cells, or intense staining in <10% of cells; IHC 2+ (equivocal) as moderate, complete circumferential, basolateral, or lateral membranous staining in ≥50% of tumor cells; IHC 3+ (positive) as intense, complete circumferential, basolateral, or lateral membranous staining in ≥50% of tumor cells. Representative IHC images for each scoring category are provided in **Supplementary Figure 1**. MMR protein status is determined by IHC detection of the four core proteins (MLH1, PMS2, MSH2, MSH6). Proficient mismatch repair (pMMR) is defined as positive expression of all four MMR proteins. dMMR is defined as the loss of nuclear expression (negative staining) in at least one of these MMR proteins (31). The cut-off for high Ki67 expression was corresponding to the median value of the cohort. Only tumor cells were scored, and the Ki67 labeling index was calculated as the percentage of Ki67-positive tumor cell nuclei out of total tumor cell nuclei examined (32). Patients were excluded if any of these clinicopathological data were incomplete.

### 2.2 Immunohistochemistry

Formalin-fixed gastric adenocarcinoma and colorectal adenocarcinoma tissue sections were dewaxed using xylene, followed by rehydration with a series of ethanol solutions. Then, antigen retrieval was accomplished with EDTA buffer (0.5 mM, pH 8.0) using a microwave for 20 minutes. Following blocking with goat serum, the sections were incubated with corresponding primary antibodies (anti-PD-L1: CST-13684s, diluted at 1:400; anti-CD4: Maixin, RMA-0620, ready-to-use; anti-CD8: Maixin, RMA-0514, ready-to-use) at 4 °C overnight. Then, after the sections were washed three times using phosphate-buffered saline (PBS). Finally, the sections were then incubated with EnVision Detection Systems Peroxidase/DAB, Rabbit/Mouse (Dako; Agilent Technologies, #K5007; ready-to-use) secondary antibodies for 30 minutes at room temperature. DAB from the aforementioned secondary staining kit was added for detection. Sections were counterstained with hematoxylin for 2 minutes at room temperature.

### 2.3 Assess the infiltration levels of CD4+ and CD8+ tumor-infiltrating lymphocytes (TILs) and the expression levels of PD-L1

Immunohistochemical staining results were independently assessed by two pathologists unaware of the clinical pathology data. When counting discrepancies exceeded 5%, re-evaluation was conducted to reach consensus. Cells were manually counted on digital images acquired with an Olympus BX-43 microscope and imaging software (Olympus cellSens). Under high magnification (400×, field area 0.2375 mm²), TILs in the tumor center and on the tumor surface, as well as those within the borders of the invasive tumor (within 0.5 mm of the tumor), were included in the enumeration (33). Ulcerated areas, ulcer bases, necrotic regions, as well as areas with high-grade intraepithelial neoplasia were excluded from the assessment. To avoid selection bias, 10 tumor-rich regions (center of tumor, CT) and 10 tumor-invasive margins (IM) were randomly selected to quantify the number of CD4 and CD8+T cells, respectively, rather than selectively choosing fields with high immune infiltration. In addition, based on the Combined Positive Score (CPS) method, the proportion of PD−L1 expression on tumor cells and within the tumor stroma was calculated separately (29, 34, 35). PD-L1 expression in tumor cells (TC)= number of PD-L1 stained cells (tumor cells)/Total number of tumor cells×100%, PD-L1 expression in tumor stroma (TS)= number of PD-L1 stained cells (immune cells)/Total number of immune cells×100%. Expression of PD-L1 in tumor cells was considered positive when membrane staining was present in more than 1% of tumor cells, while as for the PD-L1 expression in TILs, either membrane or cytoplasm staining of more than 1% TILs were considered positive (36, 37).

### 2.4 Statistical analysis

For paired continuous data, normality was tested using the Shapiro-Wilk test. The paired t-test was used for normally distributed data, and the Wilcoxon signed-rank test for non-normal data. Correlation analysis was performed using Pearson’s and Spearman’s rank correlation analysis. All statistical analyses were conducted using SPSS V.26.0 (Chicago, Illinois, USA) or GraphPad Prism V.9 (La Jolla, California, USA) software. Statistical significance was set at p <0.05. The immune cell number between biopsy and resection were corrected by Benjamini-Hochberg method for multiple comparisons.

## 3 Results

### 3.1 Clinicopathologic data of patients with gastric adenocarcinoma and colorectal adenocarcinoma patients

There were 38 patients with gastric adenocarcinoma, the age range was 37–79 years old, the median age was 61 years old, and the male-to-female ratio was 2.8:1. TNM staging (8th edition): 4 cases of T1, 0 cases of T2, 30 cases of T3, 4 cases of T4, 6 cases of N0, 10 cases of N1, 14 cases of N2, 8 cases of N3. Using 40% as the cutoff value, the low Ki67 expression group (≤40%) accounted for 57.89%, while the high expression group (>40%) accounted for 42.11%. 35 (92.11%) showed HER2 expression 0, while 3 (7.89%) showed HER2 expression 1 +. Patients were divided into two groups based on MMR status: 6 patients (15.78%) with dMMR and 32 patients (84.22%) with pMMR. Gastric sinuses ventriculi was present in 78.95% of tumors (30/38), and non-sinuses ventriculi was present in 21.05% of cases (8/38). 57.89% of cases had a tumor size <5cm (22/38), and 42.11% had a tumor size ≥5cm (16/38). 63.16% of cases had lymphovascular invasion (24/38), and 47.37% had neurovascular infiltration (18/38). 84.21% of the cases were treated with postoperative chemoradiotherapy (32/38). See **Table 1** for details.

**Table 1:** General clinicopathologic data of 38 patients with gastric adenocarcinoma.

Variables	Value
Age, median (range)	61 (37-79)
Sex, n (%)	
Male	28 (73.68)
Female	10 (26.31)
Pathologic tumor (T)/nodes (N) stage, n (%)	
T1	4 (10.53)
T2	0
T3	30 (78.94)
T4	4 (10.53)
N0	6 (15.79)
N1	10 (26.32)
N2	14 (36.84)
N3	8 (21.05)
Ki-67 expression, n (%)	
≤40%	22 (57.89)
>40%	16 (42.11)
HER2 expression, n (%)	
0	35 (92.11)
1+	3 (7.89)
2+	0
3+	0
MMR status, n (%)	
dMMR	6 (15.78)
pMMR	32 (84.22)
Location of tumor, n (%)	
Sinuses ventriculi	30 (78.95)
Non-sinuses ventriculi	8 (21.05)
Tumor size, cm	5.5 ± 4
Tumor size, n (%)	
<5.0	22 (57.89)
≥5.0	16 (42.11)
Lymphovascular invasion, n (%)	
Present	24 (63.16)
Absent	14 (36.84)
Perineural invasion, n (%)	
Present	18 (47.37)
Absent	20 (52.63)
Treatment, n (%)	
Postoperative chemoradiotherapy	32 (84.21)
No	6 (15.79)

Colorectal adenocarcinoma cohort comprised 40 patients, the age range was 25–87 years old, the median age was 64 years old, and the female to male ratio was 1:1.1. TNM staging (8th edition): 0 cases in the T1 stage, 5 cases in the T2 stage, 35 cases in the T3 stage, 15 cases in the N0 stage, 16 cases in the N1 stage, and 9 cases in the N2 stage. Using 50% as the cutoff value, the low Ki67 expression group (≤50%) accounted for 30%, while the high expression group (>50%) accounted for 70%. 18 (45%) showed HER2 expression 0, while 22 (55%) showed HER2 expression 1 +. Patients were divided into two groups based on MMR status: 7 patients (17.5%) with dMMR and 33 patients (82.5%) with pMMR. Tumors were located in the colon in 19 patients and in the rectum in 21 patients. 40% of cases had a tumor size <5cm (16/40), and 60% had a tumor size ≥5cm (24/40). 27.5% of cases had lymphovascular invasion (11/40), 15% of cases had perineural invasion (6/40). 82.5% of cases were treated with postoperative chemoradiotherapy (33/40). See **Table 2** for details.

**Table 2:** General clinicopathologic data of 40 patients with colorectal adenocarcinoma.

Variables	Value
Age, median (range)	64 (25-87)
Sex, n (%)	
Male	21 (52.5)
Female	19 (47.5)
Pathologic tumor (T)/nodes (N) stage, n (%)	
T1	0
T2	5 (12.5)
T3	35 (87.5)
N0	15 (37.5)
N1	16 (40)
N2	9 (22.5)
Ki-67 expression, n (%)	
≤50%	12 (30)
>50%	28 (70)
HER2 expression, n (%)	
0	18 (45)
1+	22 (55)
2+	0
3+	0
MMR status, n (%)	
dMMR	7 (17.5)
pMMR	33 (82.5)
Location of tumor, n (%)	
Colon	19 (47.5)
Rectum	21 (52.5)
Tumor size, cm	5.5 ± 4.0
Tumor size, n (%)	
<5.0	16 (40)
≥5.0	24 (60)
Lymphovascular invasion, n (%)	
Present	11 (27.5)
Absent	29 (72.5)
Perineural invasion, n (%)	
Present	6 (15)
Absent	34 (85)
Treatment, n (%)	
Postoperative chemoradiotherapy	33 (82.5)
No	7 (17.5)

### 3.2 Differences and correlation analysis of TILs and PD-L1 expression in biopsy specimens versus resected specimens of gastric adenocarcinoma

**Figure 1:**
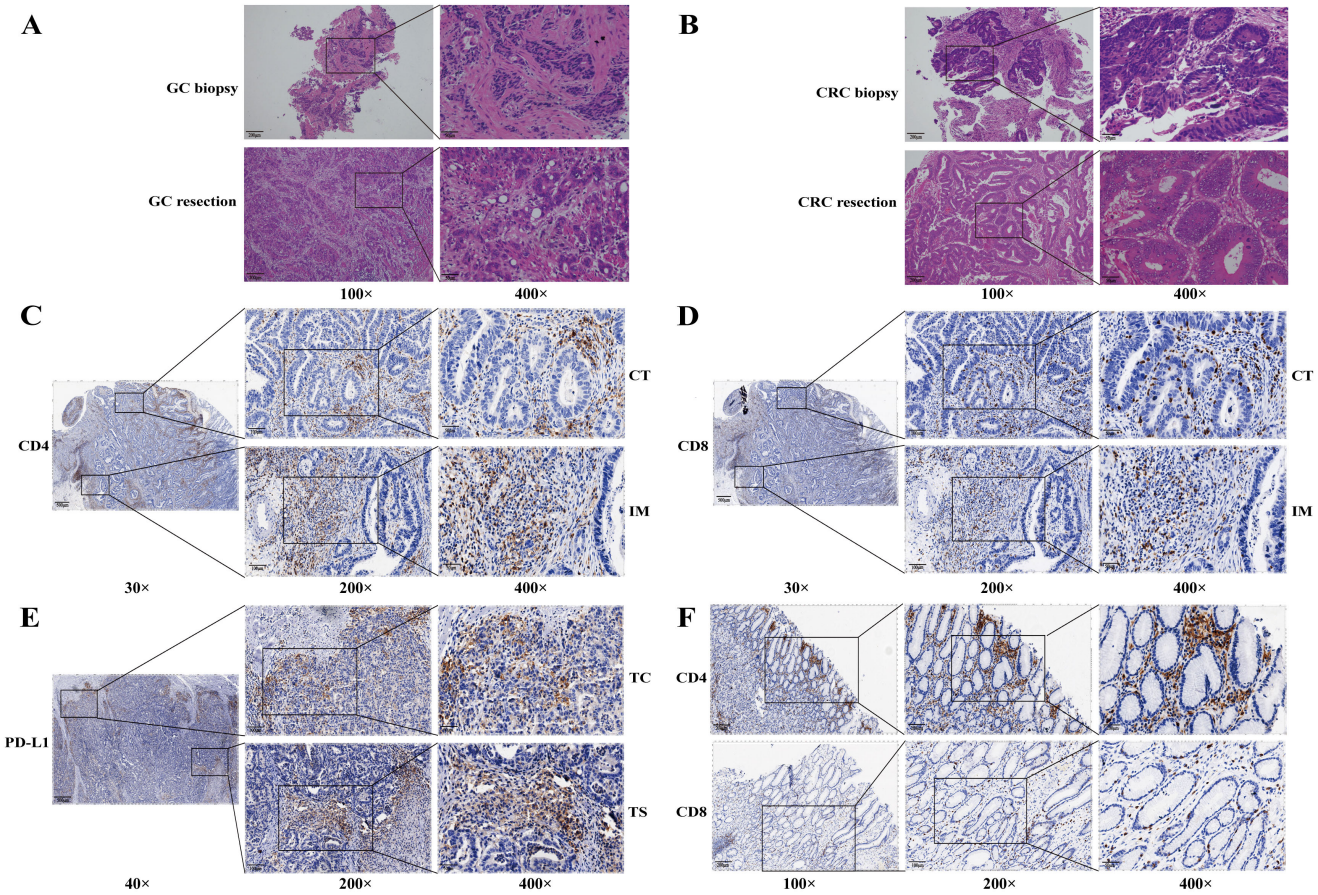
CD4+, CD8+ TILs and PD-L1 marker expression in gastric adenocarcinoma and colorectal adenocarcinoma. HE staining of tissue sections: biopsy and resection specimens of gastric and colorectal adenocarcinomas **(A, B)**. CD4+ and CD8+ immunohistochemical staining in the center of tumor and invasive margin of gastric and colorectal adenocarcinomas **(C, D)**. PD-L1 immunohistochemical staining in tumor cells and tumor stroma of gastric and colorectal adenocarcinomas **(E)**. CD4+ and CD8+ immunohistochemical staining in paracancerous tissues of gastric and colorectal adenocarcinomas **(F)**.

**Figure 2:**
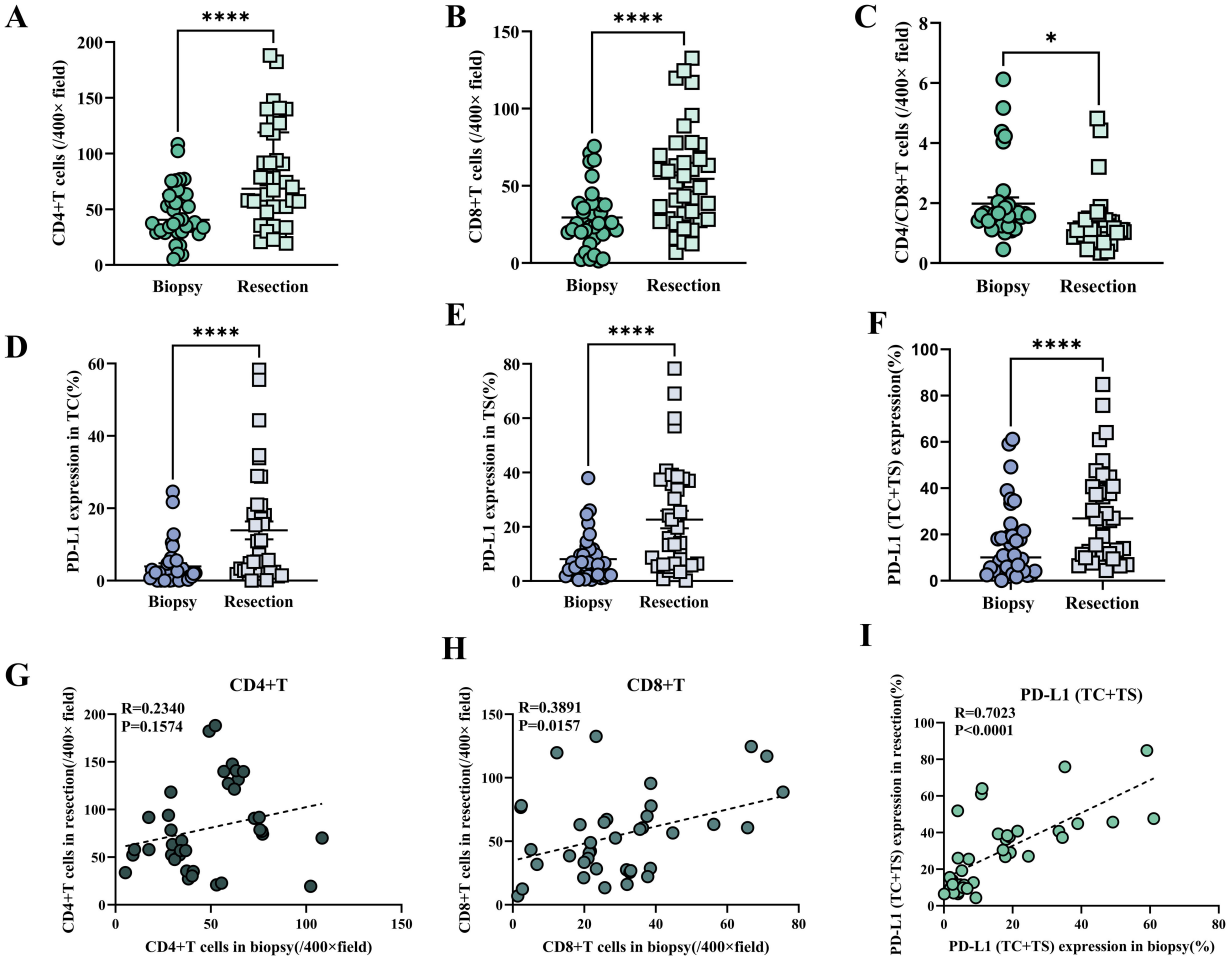
Differences and correlation analysis in TILs and PD-L1 expression in gastric adenocarcinoma biopsy specimens and resection specimens. Comparison of CD4+ T cells and CD8+ T cells densities in biopsy and resection specimens of gastric adenocarcinoma **(A, B)**. Comparison of the difference in CD4+/CD8+ T cells ratio between biopsy and resection specimens **(C)**. The difference in PD-L1 expression in tumor cells (TC) between biopsy and resection specimens **(D)**. The difference in PD-L1 expression in tumor stroma (TS) between biopsy and resection specimens **(E)**. The difference in PD-L1 expression both in tumor cells and tumor stroma between biopsy and resection specimens **(F)**. Correlation analysis of CD4+ T cells, CD8+ T cells density between biopsy and resection specimens **(G, H)**. Correlation analysis of PD-L1 expression in both tumor cells and tumor stroma between biopsy and resection specimens **(I)**. Paired t-test was used for **(B-F)**; since **(A)** did not follow a normal distribution, the Wilcoxon signed−rank test was applied. *P <0.05, ****P <0.0001; Correlations were assessed using Pearson’s correlation coefficient with two-tailed tests.

To investigate the immunological microenvironmental differences between the biopsy and the resection specimen, we evaluated the density of infiltration of CD4+ T and CD8+ T cells in the center of tumor (CT) and invasive margin (IM), as well as PD-L1 expression levels in tumor cells (TC) and tumor stroma (TS) (**Figure 1**). The TME was assessed at four sites: the CT and IM of the biopsy and resection specimen. By comparing the CD4+ T and CD8+ T cells densities of collected biopsy specimens and resection specimens of 38 gastric adenocarcinoma cases (**Figures 2A, B**), It can be found that the CD4+ T cells density of resection specimens(68.55, 51.14-119) was higher than that of biopsy specimens(40.51, 29.77-63.54) (P <0.0001, **Figure 2A**). Noticeably, CD8+ T cells density was significantly higher in resection specimens(54.56 ± 5.294) than in biopsy specimens(29.45 ± 3.071) in gastric adenocarcinoma (P <0.0001, **Figure 2B**). The CD4+/CD8+ T cell ratio in biopsy specimens(1.977 ± 0.2113) was significantly higher than in surgically resected specimens(1.327 ± 0.1506) (P = 0.0155, **Figure 2C**). PD-L1 expression in the tumor cells of gastric adenocarcinoma resection specimens(13.88 ± 2.467%) was significantly higher than in biopsy specimens(3.918 ± 0.894%) (P <0.0001, **Figure 2D**). Similarly, PD-L1 expression in the tumor stroma of resection specimens(22.62 ± 3.235%) was also significantly higher than in biopsy specimens(8.025 ± 0.319%) (P <0.0001, **Figure 2E**). Total PD-L1 expression was significantly higher in surgical resection specimens(29.20 ± 3.296%) than in biopsy specimens(16.00 ± 2.584%) (P <0.0001, **Figure 2F**). By analyzing the densities of CD4+ and CD8+ T cells, as well as the total PD-L1 expression both in tumor cells and the tumor stroma, in biopsy and resected specimens from 38 cases of gastric adenocarcinoma, we found that there was no significant correlation between the CD4+ T cell infiltration density in biopsy and resected specimens(R = 0.2340, P = 0.1574, **Figure 2G**), but a positive correlation between CD8+ T-cell infiltration density in biopsy specimens and resected specimens(R = 0.3891, P = 0.0157, **Figure 2H**), and there was a significant positive correlation between total PD-L1 expression in biopsy specimens and resected specimens (R = 0.7023, P <0.0001, **Figure 2I**).

Following the assessment of differences between specimen types, we further investigated the interrelationships among these immune markers. By bivariate correlation analysis of the expression of PD-L1, CD4+ T and CD8+ T cells in gastric adenocarcinoma (**Table 3**), there was a positive correlation between the infiltration density of CD4+ T cells in biopsy specimens and that of CD8+ T cells in biopsy specimens (R = 0.710, P = 0.001), and a positive correlation between the infiltration density of CD4+ T cells in resection specimens and that of CD8+ T cells in resection specimens (R = 0.640, P = 0.001). The density of CD8+ T cell infiltration in surgical resection specimens was positively correlated with PD-L1 expression in both biopsy specimens (R = 0.345, P = 0.034) and surgical resection specimens (R = 0.418, P = 0.009).

**Table 3:** Correlation analysis of PD-L1, CD4+ and CD8+ T cells of gastric adenocarcinoma patients.

Variables		Statistic	CD4		CD8		PD-L1	
			Biopsy	Resection	Biopsy	Resection	Biopsy	Resection
CD4	Biopsy	Spearmancorrelation	1.000	0.271	0.710**	0.220	-0.071	0.057
		Sig. N	38	0.100 38	0.001 38	0.185 38	0.671 38	0.734 38
	Resection	Spearmancorrelation	0.271	1.000	-0.080	0.640**	0.145	0.052
		Sig. N	0.100 38	38	0.746 38	0.001 38	0.384 38	0.756 38
CD8	Biopsy	Spearmancorrelation	0.710**	0.215	1.000	0.124	0.066	0.060
		Sig. N	0.001 38	0.195 38	38	0.460 38	0.694 38	0.721 38
	Resection	Spearmancorrelation	0.220	0.640**	-0.080	1.000	0.345*	0.418**
		Sig. N	0.185 38	0.001 38	0.746 38	38	0.034 38	0.009 38
PD-L1	Biopsy	Spearmancorrelation	-0.071	0.145	0.338	0.345*	1.000	0.715**
		Sig. N	0.671 38	0.384 38	0.157 38	0.034 38	38	0.001 38
	Resection	Spearmancorrelation	0.057	0.052	0.109	0.418**	0.715**	1.000
		Sig. N	0.734 38	0.756 38	0.658 38	0.009 38	0.001 38	38

Table entries are Spearman’s rho. Two-tailed test. *P <0.05, **P <0.01. Sig, two-tailed p-value; N, sample size. All variables analyzed in this table are continuous data.

### 3.3 Differences and correlation analysis of TILs and PD-L1 expression in biopsy specimens versus resected specimens of colorectal adenocarcinoma

**Figure 3:**
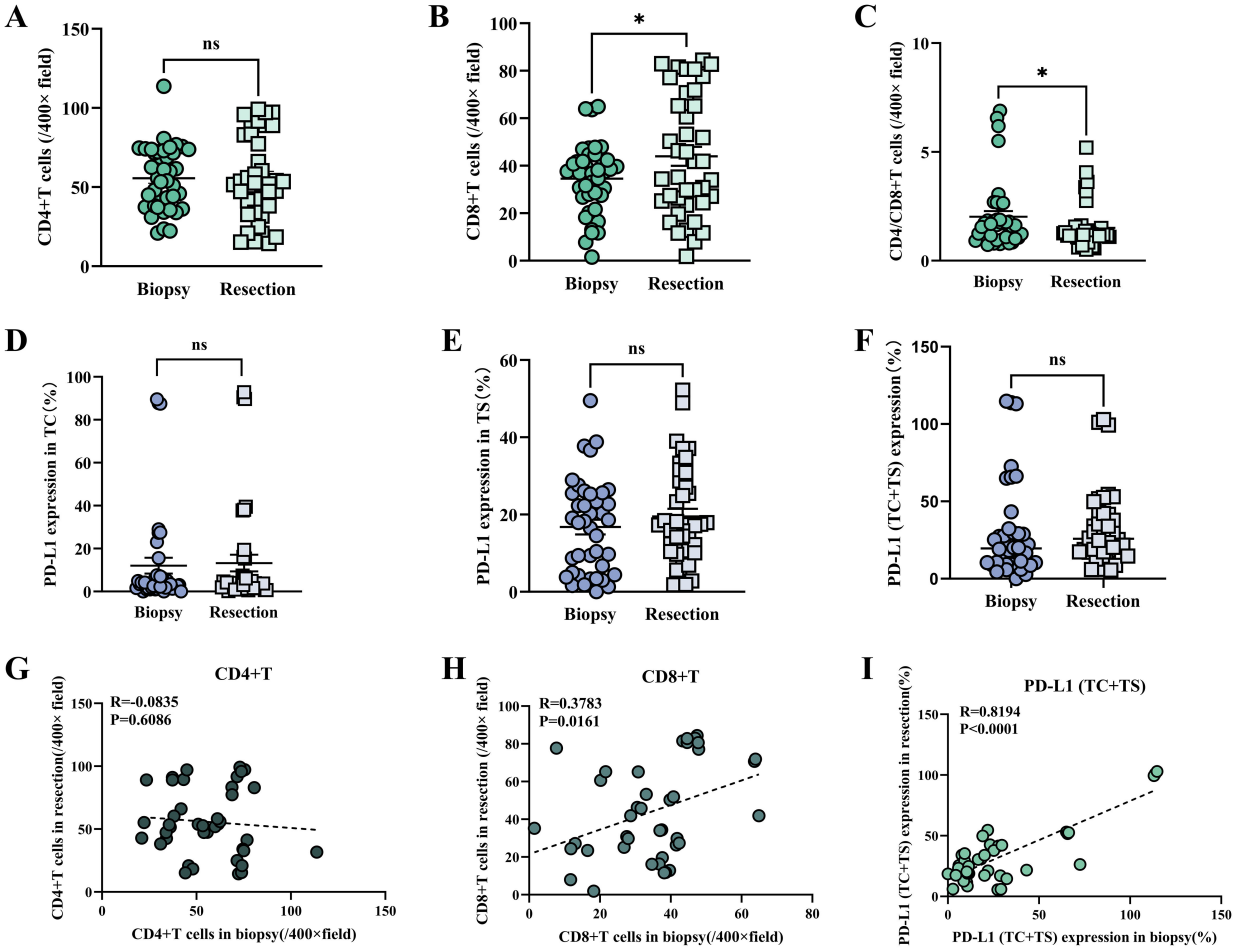
Differences and consistency in TILs and PD-L1 expression in colorectal adenocarcinoma biopsy specimens and resection specimens. Comparison of CD4+ T cells, CD8+ T cells density between colorectal adenocarcinoma biopsy and resection specimens **(A, B)**. Comparison of the difference in CD4+/CD8+ T cells ratio between biopsy and resection specimens **(C)**. The difference in PD-L1 expression in tumor cells (TC) between biopsy and resection specimens **(D)**. The difference in PD-L1 expression in tumor stroma (TS) between biopsy and resection specimens **(E)**. The difference in PD-L1 expression in both tumor cells and tumor stroma between biopsy and resection specimens **(F)**. Correlation analysis of CD4+ T cells, CD8+ T cells density between biopsy and resection specimens **(G, H)**. Correlation analysis of PD-L1 expression in both tumor cells and tumor stroma between biopsy and resection specimens **(I)**. Paired t-test was used for **(A, C-F)**; since **(B)** did not follow a normal distribution, the Wilcoxon signed−rank test was applied. Correlations were assessed using Pearson’s correlation coefficient with two-tailed tests. *P<0.05.

Having established these immunological discordances in gastric adenocarcinoma, we next examined whether similar discrepancies were present in colorectal adenocarcinoma specimens. By comparing the biopsy specimens and resection specimens of 40 colorectal adenocarcinoma patients, it can be found that the difference between the biopsy specimens(55.54 ± 3.159) and resection specimens(55.79 ± 4.189) in terms of CD4+ T cells densities were not different (P >0.05, **Figure 3A**), the CD8+ T cells density of resection specimens (38.53, 24.60-69.33) was higher than that of biopsy specimens (37.21, 26.99-43.15) (P = 0.0353, **Figure 3B**), The CD4+/CD8+ T cell ratio in biopsy specimens(2.043 ± 0.2597) was significantly higher than in surgically resected specimens(1.519 ± 0.1689)(P = 0.0168, **Figure 3C**). There was no difference in PD-L1 expression in TC and TS in biopsy specimens and resection specimens (P >0.05, **Figures 3D–F**). By analyzing the densities of CD4+ and CD8+ T cells, as well as the total PD-L1 expression both in tumor cells and the tumor stroma, in biopsy and resected specimens from 40 cases of colorectal adenocarcinoma, we found that there was no significant correlation between the CD4+ T cell infiltration density in biopsy and resected specimens(R = -0.0835, P = 0.6086, **Figure 3G**), but a positive correlation between CD8+ T-cell infiltration density in biopsy specimens and resected specimens (R = 0.3783, P = 0.0161, **Figure 3H**), and there was a significant positive correlation between total PD-L1 expression in biopsy specimens and resected specimens (R = 0.8194, P < 0.0001, **Figure 3I**).

Further analysis of PD-L1, CD4+ T and CD8+ T cells expression in colorectal adenocarcinoma by bivariate correlation analysis (**Table 4**), the density of CD4+ T cell infiltration in resected specimens of colorectal adenocarcinoma was positively correlated with the density of CD8+ T cell infiltration (R = 0.537, P = 0.001). The density of CD8+ T cell infiltration in biopsy specimens was positively correlated with that in resection specimens (R = 0.444, P = 0.004). PD-L1 expression in surgical resection specimens were negatively correlated with CD4+ T cell infiltration in resection specimens (R = -0.381, P = 0.015) and positively correlated with CD8+ T cell infiltration density in biopsy specimens (R = 0.409, P = 0.009).

**Table 4:** Correlation analysis of PD-L1, CD4+ T and CD8+ T cells of colorectal adenocarcinoma patients.

Variables		Statistic	CD4		CD8		PD-L1	
			Biopsy	Resection	Biopsy	Resection	Biopsy	Resection
CD4	Biopsy	Spearmancorrelation	1.000	-0.082	-0.004	-0.262	0.271	0.169
		Sig. N	40	0.615 40	0.983 40	0.102 40	0.091 40	0.298 40
	Resection	Spearmancorrelation	0.082	1.000	0.108	0.537**	-0.308	-0.381*
		Sig. N	0.615 40	40	0.507 40	0.001 40	0.053 40	0.015 40
CD8	Biopsy	Spearmancorrelation	-0.004	0.108	1.000	0.444**	0.095	0.409**
		Sig. N	0.983 40	0.507 40	40	0.004 40	0.561 40	0.009 40
	Resection	Spearmancorrelation	-0262	0.537**	0.444**	1.000	-0.313*	0.136
		Sig. N	0.102 40	0.001 40	0.004 40	40	0.049 40	0.403 40
PD-L1	Biopsy	Spearmancorrelation	0.271	-0.308	0.095	-0.313*	1.000	0.462**
		Sig. N	0.091 40	0.053 40	0.561 40	0.049 40	40	0.003 40
	Resection	Spearmancorrelation	0.169	-0.381*	0.409**	0.136	0.462**	1.000
		Sig. N	0.298 40	0.015 40	0.009 40	0.403 40	0.003 40	40

Table entries are Spearman’s rho. Two-tailed test. *P <0.05, **P <0.01. Sig, two tailed p-value; N, sample size. All variables analyzed in this table are continuous data.

**Figure 4:**
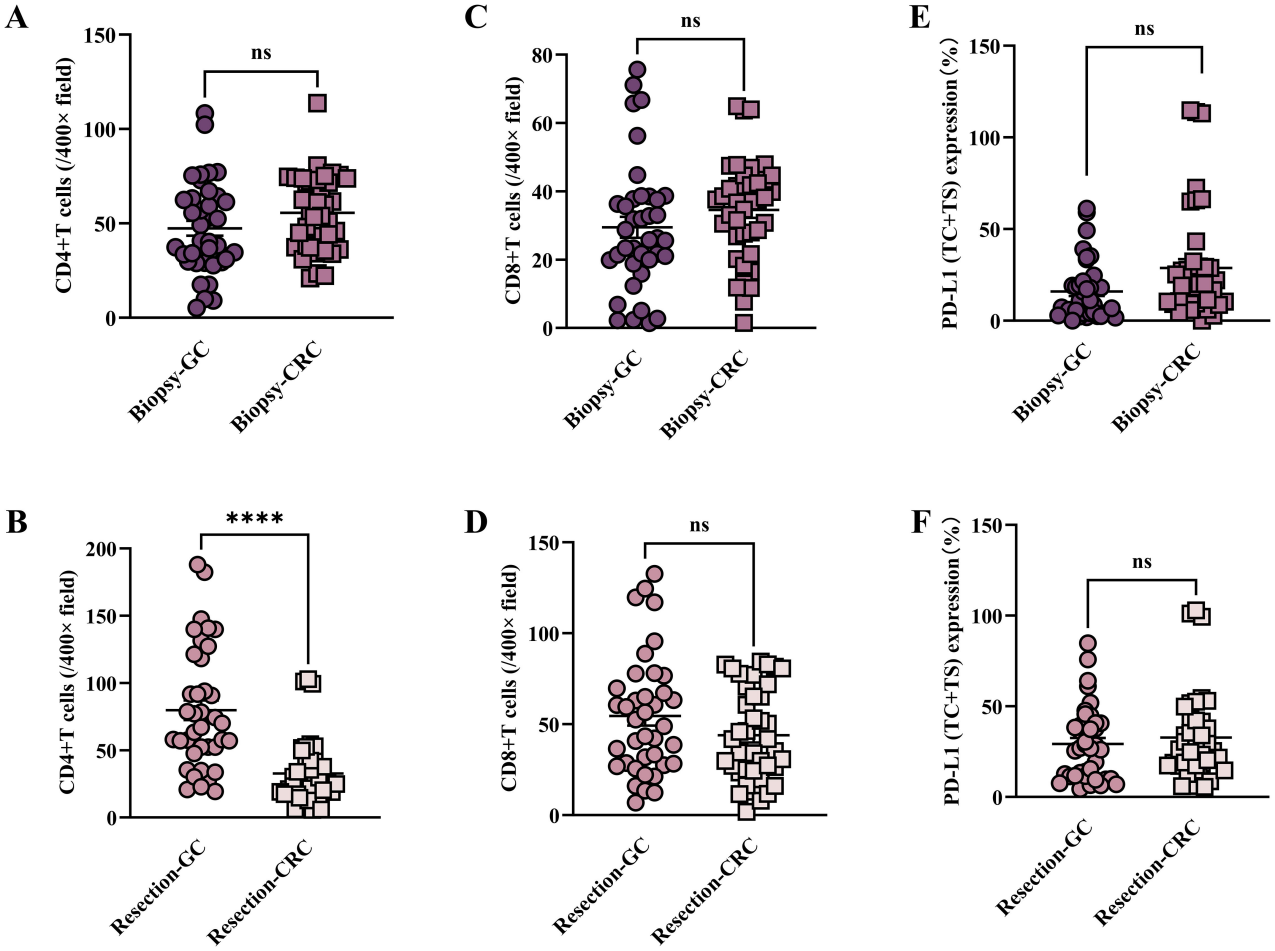
Differences in CD4+ and CD8+ T cells infiltration and PD-L1 expression between gastric adenocarcinoma and colorectal adenocarcinoma. Differences in CD4+ T cells infiltration density between gastric adenocarcinoma and colorectal adenocarcinoma **(A, B)**. Differences in CD8+ T cells infiltration density between gastric adenocarcinoma and colorectal adenocarcinoma **(C, D)**. Differences in PD-L1 expression both in tumor cells and tumor stroma between gastric adenocarcinoma and colorectal adenocarcinoma **(E, F)**. Paired t-test was used for **(B, D–F)**; since **(A, C)** did not follow a normal distribution, the Wilcoxon signed−rank test were applied. ****P <0.0001.

To determine whether the observed immune patterns were cancer-type specific, we directly compared the immune profiles of gastric and colorectal adenocarcinomas. Comparative analysis of TILs infiltration and PD-L1 expression between gastric and colorectal adenocarcinomas (**Figure 4**), it was concluded that the density of CD4+ T cells infiltration in surgically resected gastric adenocarcinoma specimens(79.74 ± 7.283) is significantly higher than that in colorectal adenocarcinoma specimens(32.75 ± 3.783) (P <0.0001, **Figure 4B**). There were no other statistically significant differences(P >0.05, **Figures 4A, C–F**).

### 3.4 Correlation of CD4+ T and CD8+ T cells infiltration in CT, IM and paracancerous tissues from surgical resection specimens

**Figure 5:**
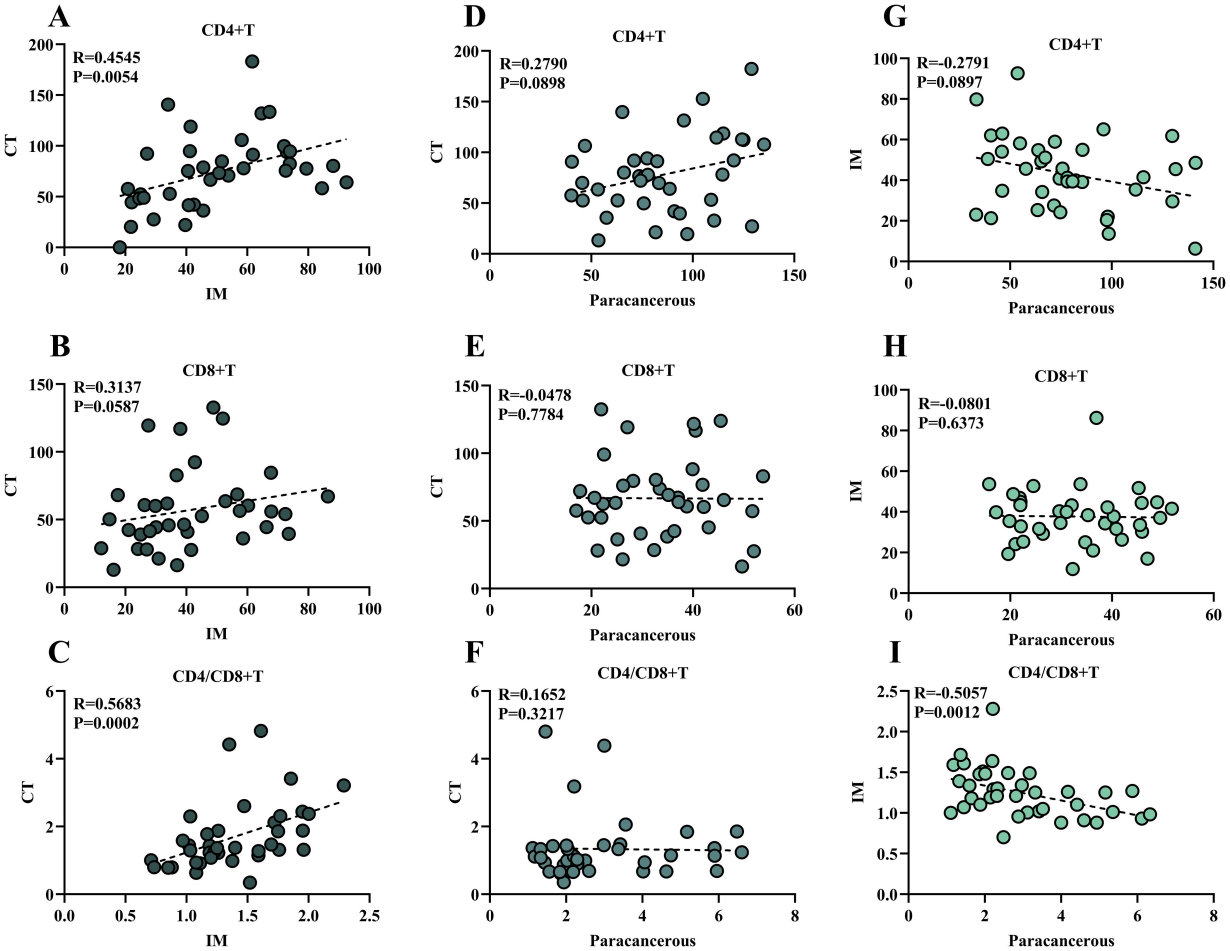
Correlation of CD4+ T and CD8+ T cells infiltration in gastric adenocarcinoma and paracancerous tissues. The correlation analysis of CD4+, CD8+ T cells infiltration and CD4+/CD8+ T-cell ratios in CT and IM **(A–C)**. The correlation analysis of CD4+, CD8+ T cells infiltration and CD4+/CD8+ T-cell ratios in CT and paracancerous tissues **(D–F)**. The correlation analysis of CD4+, CD8+ T cells infiltration and CD4+/CD8+ T-cell ratios in IM and paracancerous tissues **(G–I)**. Correlations were assessed using Pearson’s correlation coefficient with two-tailed tests.

Based on the differential patterns of TILs and PD-L1 expression observed in gastric and colorectal adenocarcinoma specimens, we further investigated the distribution of immune cell subsets by analyzing the correlation between CD4+ T and CD8+ T cells infiltration in paired cancerous and paracancerous tissues. In gastric adenocarcinoma, there was a positive correlation between the CD4+ T cells infiltration density of CT and IM in cancer (R = 0.4545, P = 0.0054, **Figure 5A**), and the ratio of CD4+/CD8+ T cells infiltration density between CT and IM exhibited a positive correlation (R = 0.5683, P = 0.0002, **Figure 5C**). Additionally, the ratio of CD4+/CD8+ T cells infiltration density in paracancerous tissue and IM showed a negative correlation (R = -0.5057, P = 0.0012, **Figure 5I**).

**Figure 6:**
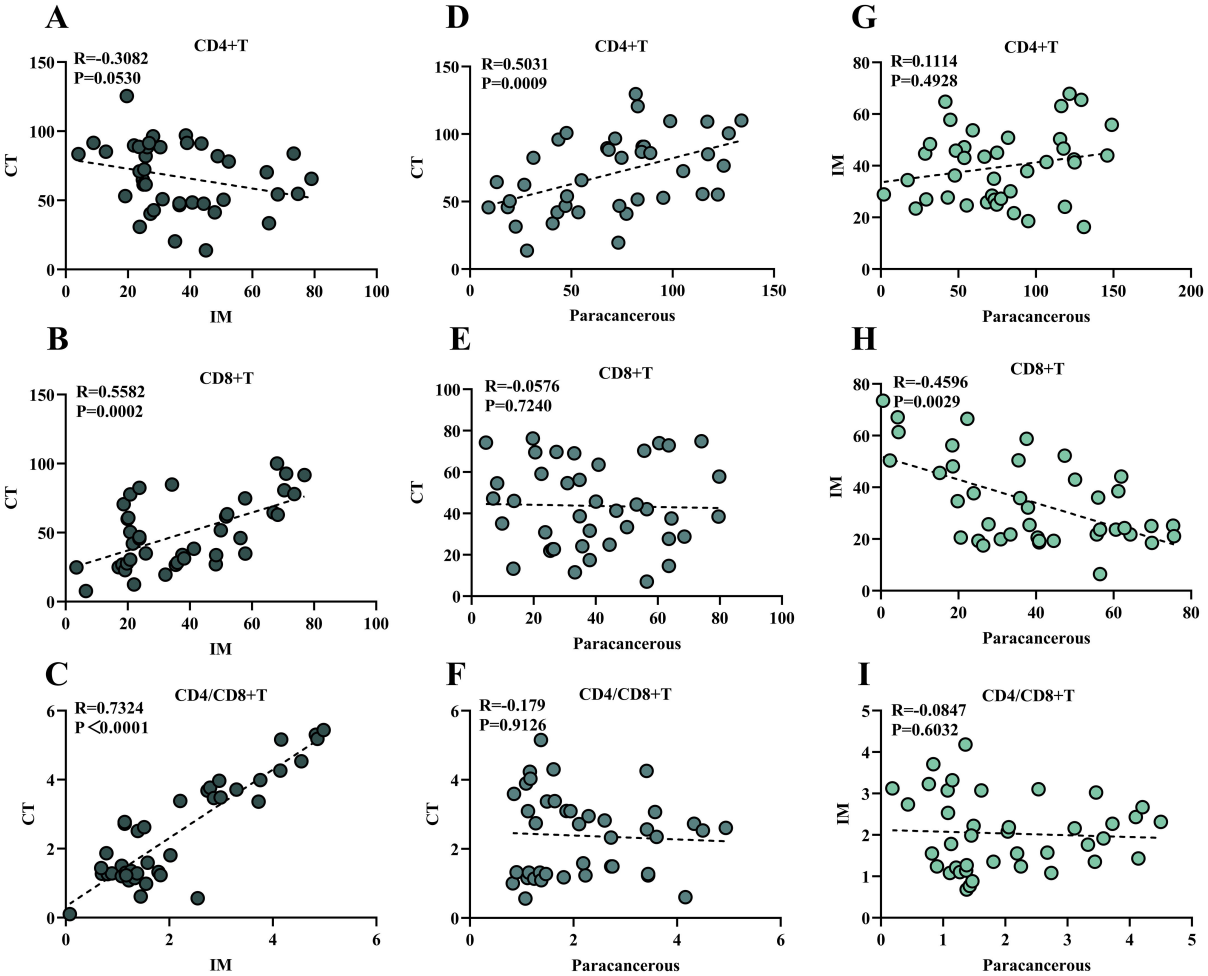
Correlation of CD4+ T cells and CD8+ T cells infiltration in colorectal adenocarcinoma and paracancerous tissues. The correlation analysis of CD4+, CD8+ T cells infiltration and CD4+/CD8+ T-cell ratios in CT and IM **(A–C)**. The correlation analysis of CD4+, CD8+ T cells infiltration and CD4+/CD8+ T-cell ratios in CT and paracancerous tissues **(D–F)**. The correlation analysis of CD4+ T, CD8+ T cells infiltration and CD4+/CD8+ T-cell ratios in IM and paracancerous tissues **(G–I)**. Correlations were assessed using Pearson’s correlation coefficient with two-tailed tests.

To determine whether the observed distribution characteristics of CD4+ T and CD8+ T cells are cancer type-specific, we performed parallel analyses on colorectal adenocarcinoma tissues. As shown in **Figure 6**, in colorectal adenocarcinoma, there was a positive correlation between the CD8+ T cells infiltration density of CT and IM in cancer (R = 0.5582, P = 0.0002, **Figure 6B**), and CD8+ T cells infiltration density in paracancerous tissues and IM showed a negative correlation (R = -0.4596, P = 0.0029, **Figure 6H**). CD4+ T cell infiltration density in the paracancerous tissue was positively correlated with that in CT (R = 0.5031, P = 0.0009, **Figure 6D**). Furthermore, the ratio of CD4+/CD8+ T cells infiltration density in CT and IM showed a positive correlation (R = 0.7324, P <0.0001, **Figure 6C**).

### 3.5 Correlation analysis of cancer, paracancerous tissues with tumor markers and clinicopathological features in gastric adenocarcinoma and colorectal adenocarcinoma patients

Having characterized the spatial distribution of CD4+ T and CD8+ T cells in gastric and colorectal adenocarcinoma tissues, we next sought to investigate whether these immune infiltration patterns correlate with key tumor markers and clinicopathological features, which may provide insights into their potential clinical relevance. The detailed results are presented in **Tables 5** and **6**. The main findings are summarized as follows.

**Table 5:** Correlation of CD4+, CD8+ T cells infiltration, PD-L1 expression and NLR with tumor markers in gastric and colorectal adenocarcinoma patients.

Variables		AFP		CA19-9		CA125		CEA	
		R	P	R	P	R	P	R	P
GC	PD-L1	-0.137	0.412	-0.135	0.419	0.007	0.965	0.111	0.506
	CD4+ T cells in cancer	-0.161	0.335	0.523*	0.026	0.137	0.413	-0.032	0.851
	CD4+T cells in paracancer	-0.2	0.23	0.139	0.404	-0.056	0.74	0.119	0.475
	CD8+T cells in cancer	-0.348*	0.035	0.012	0.942	0.102	0.548	-0.037	0.826
	CD8+T cells in paracancer	0.045	0.791	0.225	0.182	0.08	0.64	-0.203	0.228
	Ratio CD4:CD8+T cells in cancer	0.149	0.373	-0.034	0.839	0.219	0.186	0.132	0.431
	Ratio CD4:CD8+T cells in paracancer	0.137	0.421	-0.053	0.754	0.192	0.247	0.065	0.699
	Preop NLR	-0.248	0.158	-0.299	0.086	-0.169	0.339	0.15	0.399
	Postop NLR	0.01	0.956	0.02	0.912	-0.363*	0.035	-0.275	0.116
CRC	PD-L1	-0.096	0.557	-0.004	0.98	0.091	0.579	0.1	0.541
	CD4+T cells in cancer	0.067	0.681	0.167	0.303	-0.091	0.167	0.205	0.203
	CD4+T cells in paracancer	-0.444**	0.004	0.08	0.625	0.353*	0.035	0.182	0.261
	CD8+T cells in cancer	-0.032	0.895	-0.201	0.395	-0.032	0.895	0.183	0.440
	CD8+T cells in paracancer	-0.430**	0.006	0.069	0.671	0.233	0.149	0.181	0.265
	Ratio CD4:CD8+Te cells in cancer	-0.162	0.318	0.325*	0.041	0.218	0.176	0.015	0.927
	Ratio CD4:CD8+T cells in paracancer	-0.442**	0.004	0.086	0.599	0.216	0.181	0.183	0.259
	Preop NLR	-0.304	0.057	-0.036	0.825	0.198	0.22	0.111	0.494
	Postop NLR	0.041	0.804	0.233	0.148	-0.25	0.12	0.048	0.768

Preop NLR, preoperative neutrophil-to-lymphocyte ratio; Postop NLR, postoperative neutrophil-to-lymphocyte ratio. Data are Spearman’s correlation coefficients (R) with two-tailed P-values. *p < 0.05, **p < 0.01. Bold values indicate statistically significant correlations (p<0.05). "*" denotes p<0.05, and "**" denotes p<0.01.

**Table 6:** Correlation of CD4+, CD8+ T cells infiltration, PD-L1 expression and NLR with clinicopathologic features in gastric and colorectal adenocarcinoma patients.

Variables		Tumor stage		Nodes stage		Tumor diameter		Ki-67 expression		HER2 Expression		Mismatch repair protein expression	
GC	PD-L1	0.005	0.975	0.112	0.505	0.186	0.265	0.258	0.113	0.002	0.987	-0.116	0.492
	CD4+T cells in cancer	0.115	0.491	0.081	0.629	-0.012	0.944	0.136	0.448	-0.210	0.100	-0.022	0.935
	CD4+T cells in paracancer	0.214	0.196	0.198	0.234	-0.045	0.79	-0.244	0.128	0.200	0.226	-0.117	0.483
	CD8+T cells in cancer	0.218	0.195	0.032	0.851	-0.167	0.324	0.136	0.38	-0.187	0.283	-0.022	0.935
	CD8+T cells in paracancer	0.125	0.46	0.033	0.845	-0.072	0.672	-0.219	0.157	0.473**	0.009	-0.065	0.803
	Ratio CD4:CD8+T cells in cancer	0.248	0.133	0.298	0.069	0.113	0.5	0.091	0.756	-0.276	0.094	-0.165	0.527
	Ratio CD4:CD8+T cells in paracancer	0.264	0.109	0.121	0.623	-0.240	0.337	-0.252	0.119	-0.226	0.144	-0.112	0.668
	Preop NLR	0.396*	0.02	0.305	0.169	0.449*	0.011	-0.141	0.381	0.212	0.201	0.217	0.196
	Postop NLR	-0.165	0.527	0.091	0.728	0.247	0.339	-0.223	0.149	0.092	0.733	0.184	0.396
CRC	PD-L1	0.103	0.527	0.21	0.193	0.07	0.668	0.171	0.226	0.213	0.187	0.108	0.503
	CD4+T cells in cancer	0.103	0.526	0.009	0.956	0.09	0.582	-0.370*	0.019	0.174	0.224	-0.276	0.087
	CD4+T cells in paracancer	0.146	0.37	-0.047	0.771	0.261	0.134	-0.225	0.162	0.522	0.055	-0.201	0.208
	CD8+T cells in cancer	0.146	0.369	0.034	0.837	0.165	0.31	-0.025	0.879	0.251	0.146	0.224	0.166
	CD8+T cells in paracancer	0.149	0.369	-0.059	0.717	0.258	0.108	-0.228	0.158	0.026	0.930	-0.187	0.254
	Ratio CD4:CD8+T cells in cancer	0.03	0.852	0.279	0.082	-0.039	0.812	0.02	0.903	0.284	0.077	-0.342*	0.029
	Ratio CD4:CD8+T cells in paracancer	0.152	0.35	-0.047	0.771	0.269	0.093	-0.226	0.157	0.165	0.28	0.185	0.263
	Preop NLR	0.024	0.882	-0.2	0.216	0.631**	0.001	0.05	0.795	0.289	0.079	0.221	0.175
	Postop NLR	0.085	0.602	0.367*	0.021	-0.031	0.849	0.257	0.139	0.252	0.124	0.180	0.273

Preop NLR, preoperative neutrophil-to-lymphocyte ratio; Postop NLR, postoperative neutrophil-to-lymphocyte ratio. Data are Spearman’s correlation coefficients (R) with two-tailed P values.*p < 0.05, **p < 0.01. Bold values indicate statistically significant correlations (p<0.05). "*" denotes p<0.05, and "**" denotes p<0.01.

We then performed exploratory correlation analyses with tumor markers. In gastric adenocarcinoma, the density of CD4+ T cells in cancerous tissue showed a positive correlation with CA19-9 (R = 0.523, P = 0.026). In contrast, CD8+ T cell density correlated negatively with AFP (R=-0.348, P = 0.035). Postoperative neutrophil-to-lymphocyte ratio (NLR) was negatively correlated with CA125 (R=-0.363, P = 0.035). In colorectal adenocarcinoma, CD4+ T cells in paracancerous tissue correlated positively with CA125 (R = 0.353, P = 0.035). Furthermore, the densities of CD4+ T cells and CD8+ T cells in paracancerous tissue, as well as the CD4+/CD8+ T-cell ratio in paracancerous tissue, all exhibited negative correlations with AFP (R=-0.444, P = 0.004; R=-0.430, P = 0.006; and R=-0.442, P = 0.004, respectively). The CD4+/CD8+ T cells ratio in tumor tissue was positively correlated with CA19-9 (R = 0.325, P = 0.041). No other statistically significant correlations with tumor markers were observed.

Regarding clinicopathological features, preoperative NLR was positively correlated with tumor diameter in both gastric adenocarcinoma (R = 0.449, P = 0.011) and colorectal adenocarcinoma (R = 0.631, P = 0.001). In gastric cancer, preoperative NLR was also negatively correlated with tumor stage (R= -0.396, P = 0.02). In colorectal cancer, postoperative NLR was negatively correlated with nodal stage (R=-0.367, P = 0.021). Intratumoral CD4+ T cell density was negatively correlated with Ki-67 expression (R= -0.370, P = 0.019), and the intratumoral CD4+/CD8+ T-cell ratio was negatively correlated with mismatch repair protein expression (R=-0.342, P = 0.029). In gastric cancer, CD8+ T cell density in paracancerous tissue was positively correlated with HER2 expression (R = 0.473, P = 0.009). Other associations, including those with nodal stage, Ki-67, MMR status, and remaining parameters, were not statistically significant.

### 3.6 The receiver operating characteristic curve analysis of gastric adenocarcinoma, colorectal adenocarcinoma patients

**Figure 7:**
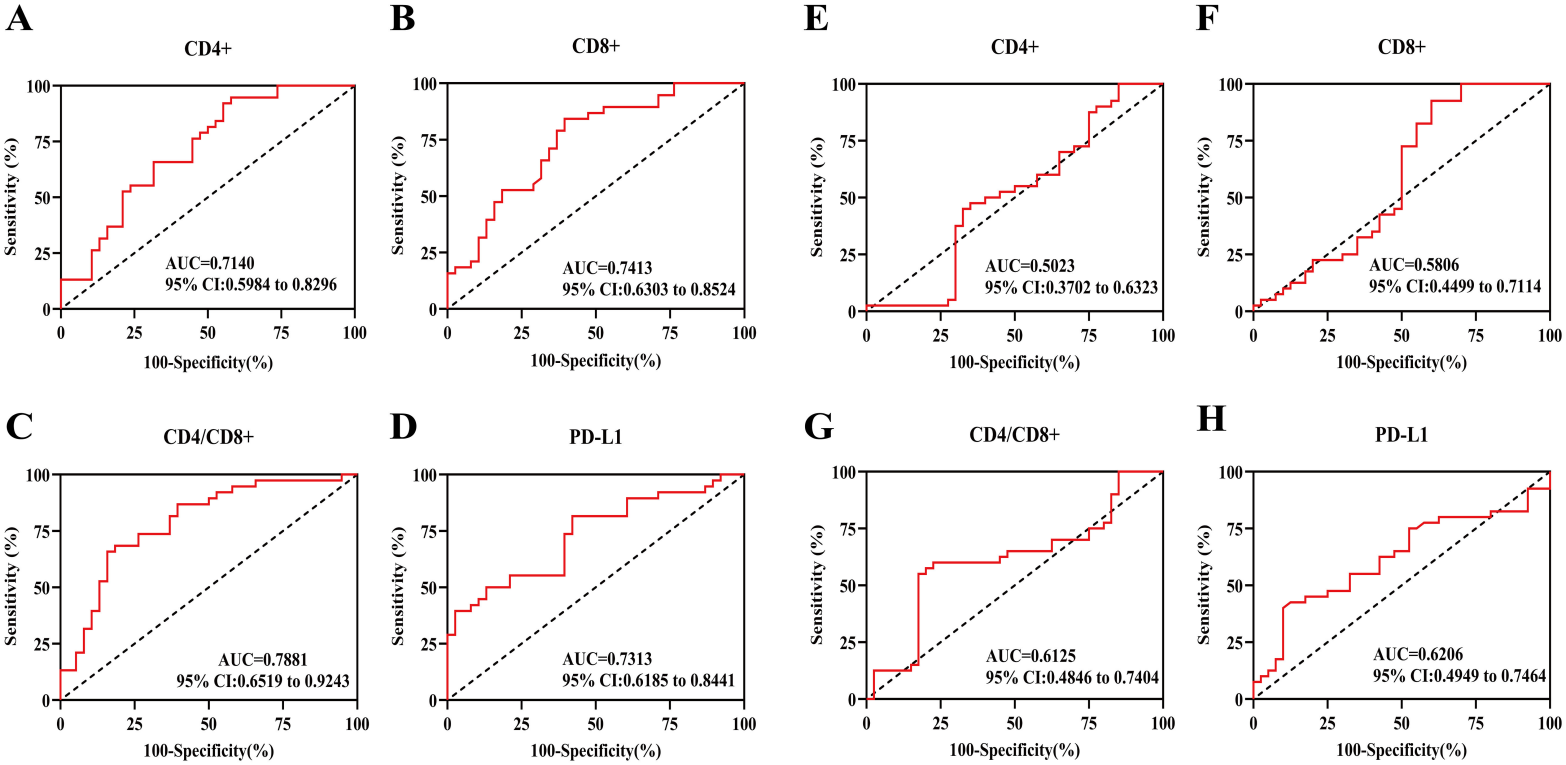
ROC curve analysis of gastric adenocarcinoma and colorectal adenocarcinoma patients. The ROC curve analysis of CD4+, CD8+ T cells, CD4+/CD8+ T-cell ratios and PD-L1 expression for gastric adenocarcinoma respectively **(A-D)**.The ROC curve analysis of CD4+, CD8+ T cells, CD4+/CD8+ T-cell ratios and PD-L1 expression for colorectal adenocarcinoma respectively **(E-H)**.

We constructed classification models for gastric and colorectal adenocarcinoma patients, employing ROC curve analysis to evaluate the predictive capability of biopsy specimen diagnostic indicators for surgical specimen gold standards (**Figure 7**). Diagnostic accuracy was measured by calculating the area under the curve (AUC). AUC interpretation followed widely accepted academic standards (30): 0.90–1.00 indicates excellent, 0.80–0.90 indicates good, 0.70–0.80 indicates fair, 0.60–0.70 indicates poor, and 0.50–0.60 indicates no diagnostic value.

For evaluating immune cell markers in the gastric adenocarcinoma tumor microenvironment, the AUC values for CD4+, CD8+ T cells, the CD4+/CD8+ T cells ratio, and PD-L1 all ranged between 0.7 and 0.8. This suggests that biopsy results and surgical findings show a certain degree of correlation, but are not entirely consistent. Regarding immunological markers in the colorectal tumor microenvironment, the ROC curves for CD4+ T cells, CD8+ T cells, CD4+/CD8+ T cells ratio, and PD-L1 expression levels all lie close to the diagonal line, with AUC values ranging between 0.5 and 0.7. This indicates poor consistency between biopsy specimens and resection specimens, suggesting that biopsy specimens have little to no surrogate value.

## 4 Discussion

This study aimed to compare the differences in CD4+ T cell and CD8+ T cell infiltration densities and PD-L1 expression levels between paired biopsy specimens and surgical resection specimens from gastric and colorectal adenocarcinomas patients, and to evaluate the clinical significance of these immune markers. Our findings indicate that the use of biopsy specimens alone is insufficient to inform immunotherapy decision-making, and that validation in larger patient cohorts is required. Accordingly, we first analyzed biopsy-resection differences and concordance, then compared immune profiles between gastric and colorectal adenocarcinomas, and finally examined correlations with tumor markers. The results demonstrated significant differences in gastric adenocarcinoma between biopsy and resection specimens for CD4+ T/CD8+ T cell infiltration density, CD4+/CD8+ T cells ratio,and PD-L1 expression levels. In gastric cancer, moderate to strong positive correlations existed between paired samples (CD8: R = 0.3891, P = 0.0157; PD-L1: R = 0.7023, P<0.0001) (**Figure 2**). Comparable moderate-to-strong correlations were also detected (CD8: R = 0.3783, P = 0.0161; PD-L1: R = 0.8194, P<0.0001) in colorectal adenocarcinoma, and CD8+ T cell infiltration density and the CD4+/CD8+ T cells ratio differed significantly (**Figure 3**). This finding contradicts the conclusions of Phillip M. Kemp Bohan et al., who reported significant differences between biopsy and resection specimens from colorectal adenocarcinoma patients in terms of CD3+ T, CD4+ T, CD8+ T cells and FOXP3+ T cells infiltration, as well as PD-L1 expression (29). Victor H. Kolzer et al. found that the density of CD8+ T cells in colorectal adenocarcinoma biopsy specimens independently predicts overall survival in patients. However, this indicator showed only a moderate correlation with matched resection specimens, and no significant correlation was observed for CD45RO+ memory T cells. This suggests that biopsy samples have a limited ability to reflect the overall T-cell infiltration status of the tumor (38). Gabriela Bindea et al. studied colorectal adenocarcinoma tissues and found that the density of CD3+, CD8+, CD45RO+ memory T cells, and B cells was significantly higher at the IM than in the CT, while these cells showed minimal infiltration in normal tissues. Concurrently, the number of macrophages in the tumor region was also significantly higher than in normal tissues. This indicates that the tumor-infiltrating front is a key site where anti-tumor T cell immune responses are most active (39).

Unlike colorectal adenocarcinoma, no studies to date have compared the tumor microenvironment between endoscopic biopsy specimens and resection specimens in gastric adenocarcinoma. However, Yonsoo Kim et al. have revealed significant histological discrepancies between biopsies and final resected specimens in early gastric adenocarcinoma. These discrepancies directly impact the selection of treatment strategies and clinical outcomes for patients (40). You Jeong Heo et al. indicated that there is a marked discrepancy in PD-L1 expression between biopsy and resection specimens in gastric adenocarcinoma, and the predictive accuracy of biopsy for the overall PD-L1 status of the tumor is limited. This difference stems in part from the co-expression of PD-L1 on both tumor cells and infiltrating immune cells (such as macrophages and lymphocytes), indirectly revealing the spatial heterogeneity of cell distribution and activity within the tumor immune microenvironment (41). Jung Soo Lee et al. demonstrated that the density of Tregs in gastric adenocarcinoma tissue was significantly higher than in adjacent normal tissue. Furthermore, elevated Treg levels have been identified as an independent adverse prognostic factor, predicting shorter recurrence-free survival and overall survival (42). Yu-Kuan Huang et al. confirmed that the density of CD163+ tumor-associated macrophages (TAMs) in gastric adenocarcinoma tumors and their transition zones is significantly higher than in normal tissues. This cell subset is associated with signaling pathways involving IL-10, IL-4, IL-3, and others, suggesting it may contribute to the formation of an immunosuppressive microenvironment through paracrine signaling or matrix regulation (43). Therefore, the tumor immune microenvironment in gastric adenocarcinoma exhibits high spatial heterogeneity. This heterogeneity directly leads to inconsistencies in the assessment of key biomarkers between small-sample biopsies and whole surgical specimens, thereby impacting the accuracy of clinical diagnosis and the optimization of treatment decisions. Therefore, the high spatial heterogeneity of the gastric adenocarcinoma tumor immune microenvironment directly leads to discrepancies in biomarker assessment between local biopsy specimens and resected specimens, thereby compromising the accuracy of clinical diagnosis and treatment.

We also compared the immune characteristics of gastric adenocarcinoma and colorectal adenocarcinoma, finding significant differences in CD4+ T cell infiltration density within the resection specimens. This finding is consistent with other studies. The immune microenvironments of gastric adenocarcinoma and colorectal adenocarcinoma exhibit distinct cancer-specific characteristics: gastric adenocarcinoma is primarily driven by TAMs and Tregs, relying on factors such as IL-6, IL-8, IL-11 and TGF-β to promote metastasis and drug resistance (44). While colorectal adenocarcinoma tends toward MDSC-mediated suppression, activating NF-κB, STAT3 pathways through pro-inflammatory factors like IL-1β, IL-17A to drive immune evasion (45). In summary, these distinct immunological characteristics between gastric adenocarcinoma and colorectal cancer not only validate the observed differences in CD4+ T cells infiltration but also provide a mechanistic basis for their divergent responses to immunotherapy.

Inflammatory microenvironment leads to T-cells infiltration to some extent and promotes tumor progression. By comparing the densities of CD4+ T and CD8+ T cells in the center of tumors and invasive margins with those in the paracancerous, and comparing the differences in CD4+/CD8+ T-cell ratio among the three, our findings indicate that the CD4+/CD8+ T cells ratio in gastric adenocarcinoma and colorectal adenocarcinoma is positively correlated between CT and the IM (**Figures 5C**, **6C**). In gastric adenocarcinoma, CD4+ T cells infiltration density significantly correlates positively between the CT and IM, while in colorectal adenocarcinoma, CD8+ T cells infiltration density significantly correlates positively between the CT and IM. Additionally, in colorectal adenocarcinomas, CD4+ T cells infiltration density in paracancerous tissue was significantly positively correlated with CT. By analyzing the correlation of cancer and paracancerous tissues with tumor markers and clinicopathological features, here we found that the density of CD4+ T cells infiltration in colorectal adenocarcinoma tissues was negatively correlated with Ki-67 expression (**Table 6**). The CD4+/CD8+ T cells ratio was also negatively correlated with mismatch repair protein expression (**Table 6**), the higher the CD4+ T-cell density, the lower the Ki-67 expression, the lower the malignancy of the tumors, and the more likely to have mismatch repair-deficient expression and unstable microsatellite expression. Ki-67 is highly expressed in malignant cells but is scarcely detectable in normal cells, making it a promising therapeutic target for cancer treatment (46). Therefore, its expression level is closely associated with tumor cell proliferation and is widely used as a proliferation marker in pathological assessment (47). Patients with higher cancer CD4+/CD8+ T-cell ratios have deficient mismatch repair protein expression and are more likely to have microsatellite instability. This is supported by the study of Greco, L. et al., who concluded that there was a significant increase in CD8+ T cells infiltration and a lower CD4+/CD8+ T-cell ratios in MSI-H tumors, which correlated with improved patient prognosis (48).

By analyzing the correlation between TILs and tumor markers as well as clinicopathological features in cancerous and paracancerous tissues, our results showed that the density of CD4+ T cells in gastric adenocarcinoma tissues were moderately positively correlated with CA19-9 (**Table 5**). As we known, CA19–9 is mainly used for prognostic judgment of pancreatic cancer, but it is not a specific biomarker, its expression has also been used in gastric adenocarcinoma. Numerous studies have shown that the high level of CA19–9 depends on the degree of gastric adenocarcinoma staging. In the advanced stage of gastric adenocarcinoma, CA19–9 can be used for prediction and diagnosis, which is important for the detection of recurrence and metastasis and post-treatment follow-up (49). Notably, this positive correlation was observed exclusively in cancerous tissue and was absent in matched adjacent non-cancerous tissue, highlighting its tumor specificity. This suggests that elevated CA19–9 levels are closely associated with an immunologically active and aggressive tumor microenvironment, potentially driven by intrinsic tumor signaling pathways that simultaneously promote lymphocyte recruitment and the development of a metastatic phenotype. Clinically, patients exhibiting high TIL density alongside elevated CA19–9 levels may represent a distinct tumor subtype. Exploring combination regimens of immune checkpoint inhibitors with systemic therapeutics for such patients warrants further investigation as a promising therapeutic direction. Furthermore, consistent with other studies, preoperative NLR in gastric adenocarcinoma and colorectal adenocarcinoma both showed a positive correlation with tumor diameter (**Table 6**), suggesting that a systemic inflammatory response could encourage tumor growth. High NLR is significantly associated with poor prognosis in a variety of tumors (e.g., colorectal, lung, breast, and gastric adenocarcinomas). Patients with high NLR had significantly shorter overall survival and progression-free survival. NLR can be used as an independent predictor of prognosis in gastric adenocarcinoma patients (50).

To comprehensively evaluate the surrogate value of biopsy specimens, this study constructed classification models for gastric adenocarcinoma and colorectal adenocarcinoma patients, respectively, to analyze the predictive performance of CD4+ T cells, CD8+ T cells, the CD4+/CD8+ T-cell ratios, and PD-L1. We observed a discordance between statistical significance and diagnostic performance. In gastric cancer, CD4, CD8 and PD-L1 expression was markedly elevated in resection specimens relative to biopsies (all P<0.05). Moderate to strong positive correlations existed between paired samples (CD8: R = 0.3891, P = 0.0157; PD-L1: R = 0.7023, P<0.0001). ROC analysis showed fair accuracy (AUC 0.7-0.8), indicating a consistent systematic expression rise largely due to tumor spatial heterogeneity and limited biopsy sampling. For colorectal cancer, CD4 and PD-L1 levels showed no significant inter-specimen differences. Comparable moderate-to-strong correlations were also detected (CD8: R = 0.3783, P = 0.0161; PD-L1: R = 0.8194, P<0.0001), whereas poor ROC consistency (AUC 0.5-0.7) revealed low sample concordance. Though group-level expression was similar, biopsies poorly predicted individual resection tissue profiles, representing unsystematic tumor heterogeneity. Such traits originate from severe intratumoral heterogeneity and scattered immune infiltration, leading to distinct individual expression variation and unsatisfactory biopsy predictive capacity. This is consistent with the study by Zhang et al., who also reported the tumor heterogeneity of colorectal cancer (29). Therefore, under current research conditions, it cannot be demonstrated that biopsy specimens can replace surgical resection specimens for assessing CD4+ T, CD8+ T, CD4+/CD8+ T-cell ratios, or PD-L1 expression.

Despite the limited sample size (38 paired gastric adenocarcinoma cases and 40 paired colorectal adenocarcinoma cases), this this limitation accurately reflects the practical challenges of obtaining matched specimens from newly diagnosed patients who have not undergone preoperative chemoradiotherapy. We employed a rigorous matching design, comparing two types of specimens from the same patient to control for inter-individual heterogeneity. This enhanced statistical power and increased the clinical credibility of observing differences within the limited sample. For the gastric adenocarcinoma cohort, with 38 paired samples and α = 0.05, the study had approximately 80% power to detect a medium-to-large effect size (Cohen’s d ≥ 0.65) for paired comparisons. For the colorectal adenocarcinoma cohort, with 40 paired samples and α = 0.05, the study had approximately 80% power to detect a medium-to-large effect size (Cohen’s d ≥ 0.64). Therefore, this study provides important preliminary evidence and a clear research framework for understanding the representativeness of biopsy specimens across different gastrointestinal tumors. Based on these findings, further validation is required through expanded sample sizes and the inclusion of a broader range of immune cell markers to comprehensively explore the distribution differences of distinct immune cell subpopulations between biopsy and surgical specimens and their clinical significance.

## 5 Conclusion

This study reveals significant discrepancies in the immune microenvironment between paired biopsy and resection specimens, with notable differences between gastric and colorectal adenocarcinomas. In gastric adenocarcinoma, resection specimens showed higher CD4+ and CD8+ T cell infiltration and PD-L1 expression than biopsies, whereas only CD8+ T cells differed in colorectal adenocarcinoma. Resection specimens capture spatial heterogeneity across tumor regions, which biopsy samples from a single site may miss. Thus, relying solely on pretreatment biopsies for immune assessment warrants caution, and biopsies should not substitute for resected tissue. CD4+/CD8+ ratios correlated positively between tumor center and invasive margin in both cancers, and TILs were significantly associated with tumor markers (CA19–9 in gastric, CA125 in colorectal adenocarcinoma). These findings provide insights for refining treatment and prognosis from an immune perspective. Larger studies incorporating broader immune markers and dynamic monitoring are needed to validate and extend these observations.

## Data Availability

The original contributions presented in the study are included in the article/**Supplementary Material**. Further inquiries can be directed to the corresponding author.
