# Correction: Differential infiltration of CD4+ and CD8+ T cells and expression of PD-L1 in paired biopsy and resection specimens of gastric and colorectal adenocarcinomas

**DOI:** 10.3389/fonc.2026.1920220

**Published:** 2026-07-13

**Authors:** Zi-xuan Wang, Chun-mei Zhang, Yao-zhen Liu, Yu-wei Zhang, Shi-jie Tong, Qi Liu, Duo Deng, Yun Pan

**Affiliations:** 1 School of Basic Medical Sciences, Dali University, Yunnan, China; 2 Department of Pathology, First Affiliated Hospital of Dali University, Yunnan, China; 3 School of Clinical Medical, Dali University, Yunnan, China

**Keywords:** biopsy and resection, CD4+ T cells, CD8+ T cells, immune microenvironment, PD-L1

The affiliation of the first author, Zixuan Wang, was erroneously given as the “School of Clinical Medicine, Dali University,” instead of the correct affiliation, the “School of Basic Medical Sciences, Dali University.” The original version of this article has been updated.

