# Supplementary figures and images for: Differential infiltration of CD4+ and CD8+ T cells and expression of PD-L1 in paired biopsy and resection specimens of gastric and colorectal adenocarcinomas
Source: Front Oncol. 2026 Jun 22;16:1830997. doi: 10.3389/fonc.2026.1830997 (PMC13333484; doi:10.3389/fonc.2026.1830997)

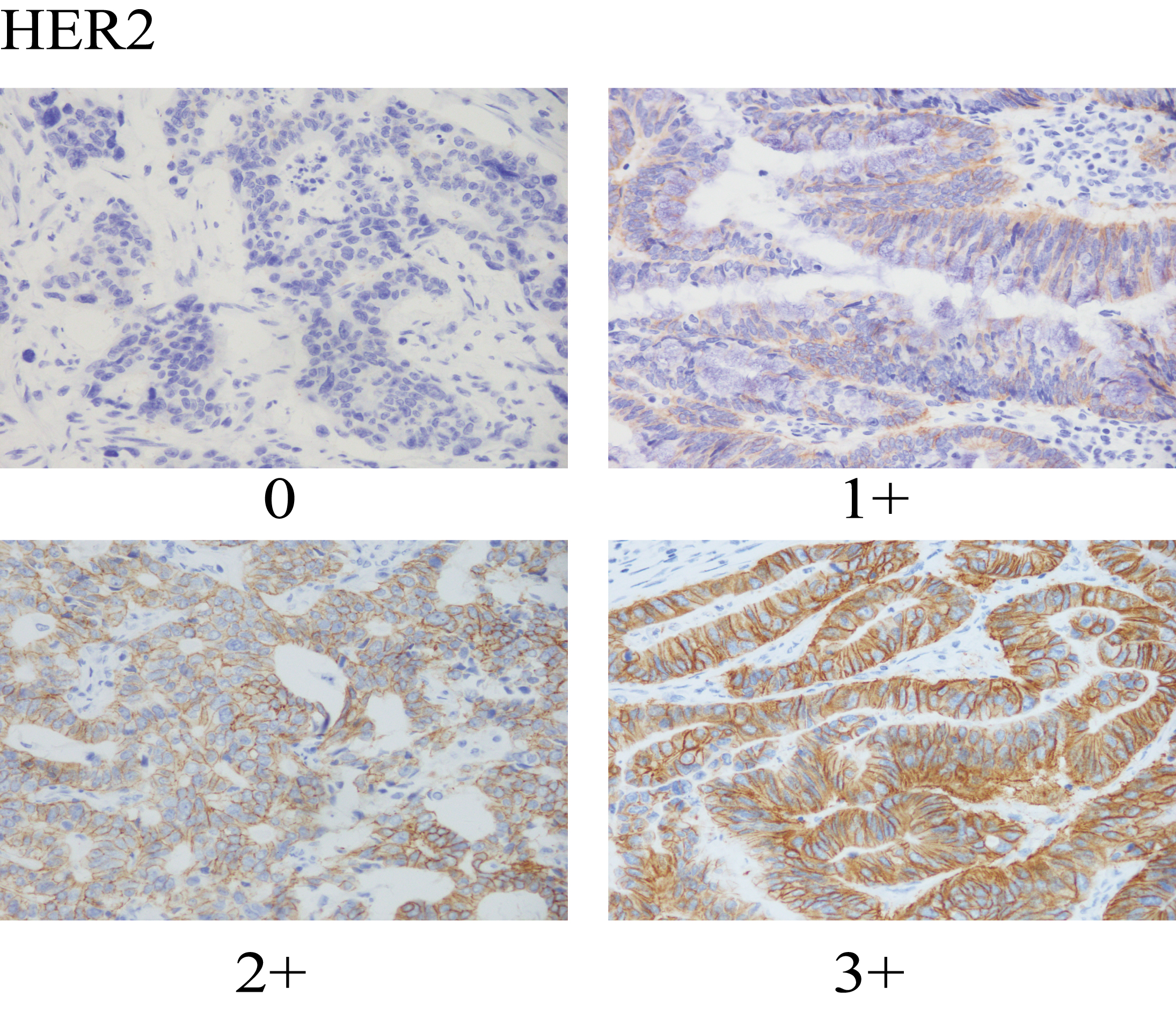

Supplement: Supplementary file 1 [file Image1.jpeg]
